# Data on corona-virus readiness strategies influencing customer satisfaction and customer behavioural intentions in South African retail stores

**DOI:** 10.1016/j.dib.2020.105818

**Published:** 2020-06-05

**Authors:** Tarisai Fritz Rukuni, Eugine Tafadzwa Maziriri

**Affiliations:** Department of Business Management, University of the Free State, Bloemfontein, South Africa

**Keywords:** Sanitised retail entrances, Sanitised retail counters, Sanitised retail shelves, Retail social distancing, Senior citizens shopping hours, Customer satisfaction, Retail covid-19 readiness, Customer behavioural intentions

## Abstract

This article presents raw inferential statistical data that determined the coronavirus readiness strategies at retail stores in South Africa and their consequences for consumer behavioural intentions. The data was collected from customers within the metropolitan area of Bloemfontein. The data were analysed using a quantitative approach. Structured questionnaires were provided to customers throughout South Africa's Bloemfontein metropolitan area. Reliability and validity were confirmed. The data was presented using Structural Equation modelling (SEM) using the Smart PLS program. The analysis of the SEM path shows estimates of the interconnectivity of the major constructs in the data. The findings from this dataset show that sanitised retail entrances, sanitised retail counters and sanitised retail shelves had a statistically significant effect on customer satisfaction with covid-19 readiness in retail stores. Furthermore, the data reveals that retail social distancing and senior citizens shopping hours had a statistically insignificant effect on customer satisfaction with covid-19 readiness. Moreover, the data reveals that customer satisfaction with covid-19 readiness strategies of retail stores also had a positive and statistically significant effect on customer behavioural intentions.

Specifications table

**Table:** 

Subject	Business and Marketing
Specific subject area	Consumer behaviour, grocery consumption, retailing
Type of data	Tables and figures
How data were acquired	Data was gathered significantly through the dissemination of questionnaires to clients inside the Bloemfontein Metropolitan region
Data format	Raw, analysed, descriptive and statistical data
Parameters for data collection	To qualify for inclusion in the sample the participant had to be customers within the Bloemfontein metropolitan area.
Description of data collection	The data was collected using self-administrated questionnaires in Bloemfontein from 344 willing customers. All the questionnaires were hard copy printouts.
Data source location	Bloemfontein, South Africa
Data accessibility	Data is included in this article

## Value of the data

• The data helps explain the impact of sanitised retail entrances, sanitised retail counters, sanitised retail shelves, retail social distancing and senior citizens shopping hours on customer satisfaction with retail covid-19 readiness and customer behavioural intentions.
• The data can be used to enlighten retail marketing managers on the importance of sanitised retail entrances, sanitised retail counters, sanitised retail shelves, retail social distancing and senior citizens shopping hours, as well as how these five variables can be beneficial to customer satisfaction with retail covid-19 readiness and customer behavioural intentions.
• The data can be used as a springboard for further discourse on how retail managers could enhance retail covid-19 readiness strategies.
• Given the topical issue of the corona virus pandemic, data presented in this data article provides retail strategies which might be utilised to eradicate the spread of the corona virus.
• The data shows the level of impact each of the variables have on each other.
• The data can be modified for use in other contexts.

## 1 Data description

**Fig. 1:**
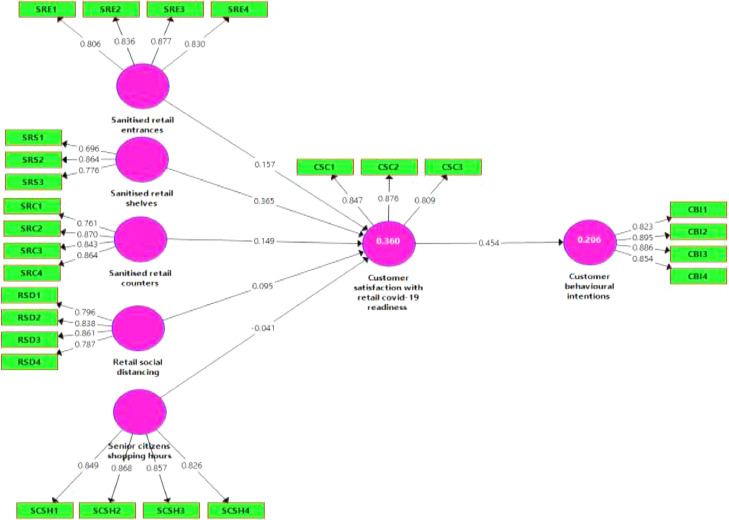
Structural model.

The data is presented through two tables and one figure. In Table 1, the accuracy analysis statistics are presented which include reliability and validity measures. Three methods, namely Cronbach's alpha test (Cronbach α), composite reliability test (CR) and average value extracted (AVE) test were used to check on the reliability of data. Table 1 below displays that all the reliability values were above the recommended value of 0.7 [4], suggesting excellent levels of internal consistency. Fig. 1, illustrates the structural model showing all the outcomes of the proposed hypotheses. Table 2 presents the sample profile showing demographic data of the participants such as gender and age. Lastly, Table 3 presents the hypotheses results. Moreover, this data article is accompanied with supplementary data files, precisely a micro-soft excel spreadsheet data set and a structured questionnaire which is divided into eight sections.

**Table 1:** Measurement accuracy assessment.

Research constructs	PLS code item	Scale item		Cronbach's alpha value	Composite reliability	Average variance extracted (AVE)	Factor loadings
		Mean	SD				
Sanitised retail entrances	SRE1	3.178	1.181	0.858	0.904	0.702	0.806
	SRE2	3.930	0.932				0.836
	SRE3	3.439	0.986				0.877
	SRE4	3.159	1.154				0.830
Sanitised retail shelves	SRS1	3.159	1.115	0.679	0.824	0.611	0.696
	SRS2	3.064	1.032				0.864
	SRS3	3.471	1.032				0.776
Sanitised retail counters	SRC1	3.274	1.104	0.856	0.902	0.698	0.761
	SRC2	3.140	1.202				0.870
	SRC3	3.873	0.949				0.843
	SRC4	3.535	1.080				0.864
Retail social distancing	RSD1	3.395	1.081	0.838	0.892	0.674	0.796
	RSD2	3.541	1.056				0.838
	RSD3	3.497	0.988				0.861
	RSD4	3.025	1.094				0.787
Senior citizens shopping hours	SCSH1	3.013	1.041	0.873	0.912	0.723	0.849
	SCSH2	3.025	1.022				0.868
	SCSH3	3.274	1.104				0.857
	SCSH4	3.140	1.202				0.826
Customer satisfaction	CSC1	3.331	0.967	0.799	0.882	0.713	0.847
	CSC2	3.089	1.055				0.876
	CSC3	3.140	1.202				0.809
Customer behavioural intentions	CBI1	3.873	0.949	0.888	0.922	0.748	0.823
	CBI2	3.535	1.080				0.895
	CBI3	3.395	1.081				0.886
	CBI4	3.541	1.056				0.854

**Table 2:** Sample profile.

	Representation
**Age**	
18–25	24%
26–35	36%
36–45	9%
46+	31%
**Total**	100%
**Gender**	
Male	45%
Female	55%
**Total**	100%

**Table 3:** Outcomes of structural equation model analysis.

Path	Hypothesis	Path coefficients (β)	T- Statistics	Decision
Sanitised retail entrances -> Customer satisfaction with retail covid-19 readiness	H1(+)	0.157	2.382	Positive and insignificant
Sanitised retail shelves -> Customer satisfaction with retail covid-19 readiness	H2(+)	0.365	5.592	Positive and significant
Sanitised retail counters -> Customer satisfaction with retail covid-19 readiness	H3(+)	0.149	2.251	Positive and significant
Retail social distancing -> Customer satisfaction with retail covid-19 readiness	H4 (-)	0.095	1.505	Positive and insignificant
Senior citizens shopping hours -> Customer satisfaction with retail covid-19 readiness	H5 (+)	−0.041	0.688	Negative and insignificant
Customer satisfaction with retail covid-19 readiness -> Customer behavioural intentions	H6 (+)	0.454	9.444	Positive and significant

## 2 Experimental design, materials and methods

The data presented was analysed using a quantitative approach. A descriptive research design was adopted to obtain the opinions of customers concerning the customer satisfaction with retail covid-19 readiness and customer behavioural intentions. A survey method was considered an appropriate data collection method because it allows for the collection of standardised data that permits the researcher to produce information for answering the how, who, what and when questions regarding the subject matter.

A total of ten retail stores were considered giving a total population of 220 000 customers in Bloemfontein, South Africa. Based on the Slovin's formula [5], the minimum sample size for this population was 401. From the 401 received questionnaires, the response rate was 85 per cent as seen by the 344 responses that were filled completely and ready for analysis. It is important to note that at least 40 customers were drawn from each of the ten retail stores. Furthermore, it is imperative to note that the researchers engaged in the data preparation process. According to Aaker, Kumar and Day [1], data preparation is regarded as a process of converting data from a questionnaire into a format that can be analysed. Furthermore, there are four phases of data preparation, namely data editing, coding, capturing and cleaning [1, 3]. These phases were employed to ensure that the data collected is complete and ready for analysing. After checking for missing values and outliers in the data, the researchers proceeded in assessing the reliability of test results. To test the data, the researchers proposed the model whereby sanitised retail entrances, sanitised retail shelves, sanitised retail counters, retail social distancing and senior citizens shopping hours were the predictor variables. Customer satisfaction with retail covid-19 readiness was the mediating variable. Moreover, customer behavioural intentions was the outcome variable. The researchers had to propose a model to test the validity of the proposed model as well as to determine if the data, which has been collected in the field, fits well with the proposed conceptual model. Table 2 displays demographic data of the participants.

### 2.1 Path model

The PLS estimation results for the structural model, path coefficients values as well as the item loadings for the research constructs are shown in Fig. 1.

The primary source of data (questionnaire) was used for collecting data from a cross section of customers within the Bloemfontein metropolitan area. The Microsoft Excel spreadsheet worksheet is used to enter all data and draw conclusions from the data obtained. The Statistical Packages for Social Sciences (SPSS) and the Smart PLS software for structural equation modelling (SEM) technique were used to code data and to run the statistical analysis [2]. Moreover, Smart PLS supports both exploratory and confirmatory research; it is robust to deviations for multivariate normal distributions and is good for a small sample size [2].

## 3 Ethical considerations

The researchers adhered to all ethical consideration during the data collection process. The researchers ensured that the respondents were well acquainted with the participation process. Moreover, Respondents were given the ability to remain anonymous and their comments reactions were dealt with in confidence.

## 4 Academic, practical and policy implications of this data article

The present data article offers implications for academicians. For instance, the data indicates that sanitised retail shelves directly influence customer satisfaction with retail covid-19 readiness in a positive and significant way as indicated by a path coefficient of (β=0.365). Therefore, for academicians in the field of retail marketing, this discovery enhances their understanding of the relationship between sanitised retail shelves and customer satisfaction with retail covid-19 readiness as this is a useful contribution to the existing literature on these two variables. On the practitioners’ side, this data article submits that retail managers can benefit from the implications of these discoveries. For example, given the robust relationship between sanitised retail entrances and customer satisfaction with retail covid-19 readiness (β=0.157), marketing managers ought to pay attention or they should put more emphasis on sanitizing their retail entrances, this will ultimately make customers satisfied and for them to perceive retailers as ready to tackle covid-19. Moreover, the present data article offers implications for policy makers who have been developing business policies that enhance tackle the issue of covid-19. Policies that exist in various retail institutions can be modified to incorporate covid-19 readiness strategies that have been presented in this data article. Thus, the discoveries obtained from the analysed data set may be used to generate new policies and assist in the revision of existing policies.

## Declaration of Competing Interest

The authors declare that they have no known competing financial interests or personal relationships which have, or could be perceived to have, influenced the work reported in this article.

## Appendix Supplementary materials

Image, application 1

Image, application 2
