# Supplementary material for: Data on corona-virus readiness strategies influencing customer satisfaction and customer behavioural intentions in South African retail stores
Source: Data Brief. 2020 Jun 5;31:105818. doi: 10.1016/j.dib.2020.105818 (PMC7274094; doi:10.1016/j.dib.2020.105818)
Supplement: Supplementary file 1 [file mmc1.docx]

**QUESTIONNAIRE**

**SECTION A: DEMOGRAPHICS**

**1.What is your age in years?**

| 18-25 | 1 |
| --- | --- |
| 26-35 | 2 |
| 36-45 | 3 |
| 46 + | 4 |

**2. What is your gender?**

| Male | 1 |
| --- | --- |
| Female | 2 |

**____________________________________________**

**SECTION B: OPINIONS SOUGHT ON RETAIL COVID-19 READINESS STRATEGIES**

In this section, please indicate the extent to which you agree or disagree with each of the following statements. You may indicate your answer by placing a cross (x) in your selected response, using the scale: (5) = Strongly Agree; (4) = Agree; (3) = Neutral; (2) = Disagree; (1) = Strongly Disagree

| **Sanitised retail entrances** | | **Strongly Agree**  **(5)** | **Agree**  **(4)** | **Neutral**  **(3)** | **Disagree**  **(2)** | **Strongly Disagree**  **(1)** |
| --- | --- | --- | --- | --- | --- | --- |
| **SRE1** | There are sanitised wipes for trollies at the retail store entrance | 5 | 4 | 3 | 2 | 1 |
| **SRE2** | Retail store personnel at the entrance encourage customers to wipe trollies | 5 | 4 | 3 | 2 | 1 |
| **SRE3** | Retail store personnel at the entrance spray customers’ hands with a sanitised spray | 5 | 4 | 3 | 2 | 1 |
| **SRE4** | Retail store personnel at the entrance wears protective clothing (i.e. face mask, gloves) | 5 | 4 | 3 | 2 | 1 |

| **Sanitised retail shelves** | | **Strongly Agree**  **(5)** | **Agree**  **(4)** | **Neutral**  **(3)** | **Disagree**  **(2)** | **Strongly Disagree**  **(1)** |
| --- | --- | --- | --- | --- | --- | --- |
| **SRS1** | The retail store keeps sanitised spray on its shelves to spray customers | 5 | 4 | 3 | 2 | 1 |
| **SRS2** | Retail store employees spray sanitized spray on customers’ hands when they reach the shelves | 5 | 4 | 3 | 2 | 1 |
| **SRS3** | Retail store employees keep spraying sanitized spray on the shelves | 5 | 4 | 3 | 2 | 1 |

| **Sanitised retail counters** | | **Strongly**  **Agree**  **(5)** | **Agree**  **(4)** | **Neutral**  **(3)** | **Disagree**  **(2)** | **Strongly Disagree**  **(1)** |
| --- | --- | --- | --- | --- | --- | --- |
| **SRC1** | There is sanitised spray is kept on the store counter | 5 | 4 | 3 | 2 | 1 |
| **SRC2** | The retail store employees spray customers’ hands at the counter | 5 | 4 | 3 | 2 | 1 |
| **SRC3** | The retail store employees keep wiping the store counter with sanitised wipes | 5 | 4 | 3 | 2 | 1 |
| **SRC4** | The retail store employees at the counter wear protective clothing (i.e. face masks, gloves) | 5 | 4 | 3 | 2 | 1 |

| **Retail social distancing** | | **Strongly Agree**  **(5)** | **Agree**  **(4)** | **Neutral**  **(3)** | **Disagree**  **(2)** | **Strongly Disagree**  **(1)** |
| --- | --- | --- | --- | --- | --- | --- |
| **RSD1** | The retail store controls the number of customers entering the store at a time | 5 | 4 | 3 | 2 | 1 |
| **RSD2** | The retail store has wide space between shelves | 5 | 4 | 3 | 2 | 1 |
| **RSD3** | The retail store has posters that encourage customers to keep at least one-meter distance when walking in the store | 5 | 4 | 3 | 2 | 1 |
| **RSD4** | The retail store encourages customers to keep at least one-meter distance when waiting on the till queue | 5 | 4 | 3 | 2 | 1 |

| **Senior citizens shopping hours** | | **Strongly Agree**  **(5)** | **Agree**  **(4)** | **Neutral**  **(3)** | **Disagree**  **(2)** | **Strongly Disagree**  **(1)** |
| --- | --- | --- | --- | --- | --- | --- |
| **SCS1** | The retail specifies shopping hours for senior citizens | 5 | 4 | 3 | 2 | 1 |
| **SCS2** | The retail store specifies the exact age groups that are regarded as senior citizens | 5 | 4 | 3 | 2 | 1 |
| **SCS3** | The retail store has a notice on the entrance that specify shopping hours for senior citizens | 5 | 4 | 3 | 2 | 1 |
| **SCS4** | The retail store accommodates customers with underlying health conditions in the shopping hours for senior citizens | 5 | 4 | 3 | 2 | 1 |

| **Customer satisfaction with retail COVID-19 readiness** | | **Strongly Agree**  **(5)** | **Agree**  **(4)** | **Neutral**  **(3)** | **Disagree**  **(2)** | **Strongly Disagree**  **(1)** |
| --- | --- | --- | --- | --- | --- | --- |
| **CS1** | Am satisfied with the sanitisation measures applied at the retail store entrance | 5 | 4 | 3 | 2 | 1 |
| **CS2** | Am satisfied with the sanitisation measures applied at the retail store shelves | 5 | 4 | 3 | 2 | 1 |
| **CS3** | Am satisfied with the sanitisation measures applied at the retail store counter | 5 | 4 | 3 | 2 | 1 |
| **CS4** | Am satisfied with the efforts made by the retail store to encourage social distancing | 5 | 4 | 3 | 2 | 1 |

| **Customer behavioural intentions** | | **Strongly Agree**  **(5)** | **Agree**  **(4)** | **Neutral**  **(3)** | **Disagree**  **(2)** | **Strongly Disagree**  **(1)** |
| --- | --- | --- | --- | --- | --- | --- |
| **CBI1** | I will buy again in this retail store | 5 | 4 | 3 | 2 | 1 |
| **CBI2** | I will refer my friends to this retail store | 5 | 4 | 3 | 2 | 1 |
| **CBI3** | I will refer my relatives to this retail store | 5 | 4 | 3 | 2 | 1 |
| **CBI4** | I will tell my friends good things about this retail store | 5 | 4 | 3 | 2 | 1 |
